# Predictors of death among TB/HIV co-infected patients on tuberculosis treatment in Sichuan, China: A retrospective cohort study

**DOI:** 10.1097/MD.0000000000032811

**Published:** 2023-02-03

**Authors:** Ni Yang, Jinge He, Jing Li, Yin Zhong, Yang Song, Chuang Chen

**Affiliations:** a Sichuan Center for Disease Control and Prevention, Chengdu, Sichuan, China.

**Keywords:** China, risk factors, survival, TB/HIV co-infection

## Abstract

*Mycobacterium tuberculosis* is the most common opportunistic infection among patients with human immunodeficiency virus (HIV) infection, and it is also the leading cause of death, causing approximately one-third of acquired immune deficiency syndrome deaths worldwide. China is on the World Health Organization's global list of 30 high-tuberculosis (TB) burden countries. The objective of this study was to evaluate the mortality rate, survival probabilities, and factors associated with death among patients with TB/HIV co-infection undergoing TB treatment in Sichuan, China. A retrospective cohort study was conducted using the Chinese National TB Surveillance System data of TB/HIV co-infected patients enrolled in TB treatment from January 2020 to December 2020. We calculated the mortality rate and survival probabilities using the Kaplan–Meier estimator, and a Cox proportional hazard model was conducted to identify independent risk factors for TB/HIV co-infection mortality. Hazard ratios and their respective 95% confidence intervals were also reported in this study. Of 828 TB/HIV co-infected patients, 44 (5.31%) died during TB treatment, and the crude mortality rate was 7.76 per 1000 person-months. More than half of the deaths (n = 23) occurred in the first 3 months of TB treatment. Overall survival probabilities were 97.20%, 95.16%, and 91.75% at 3rd, 6th, and 12th month respectively. The independent risk factors for mortality among TB/HIV co-infected patients were having extra-pulmonary TB and pulmonary TB co-infection, history of antiretroviral therapy interruption, and baseline cluster of differentiation 4 T-lymphocyte counts <200 cells/μL at the time of HIV diagnosis. Antiretroviral therapy is important for the survival of TB/HIV co-infected patients, and it is recommended to help prolong life by restoring immune function and preventing extra-pulmonary TB.

## 1. Introduction

In areas with high human immunodeficiency virus (HIV) prevalence, the number of patients with tuberculosis (TB) among people with HIV infection is increasing rapidly, posing a new challenge for TB control. TB remains the leading presenting opportunistic infection among people with HIV infection, and HIV-positive individuals are 16 to 27 times more likely to contract TB than HIV-negative individuals.^[1]^ Moreover, TB is the leading cause of death from infectious diseases globally, causing approximately 214,000 deaths among HIV-positive people globally in 2020 (up from 209,000 in 2019) according to the World Health Organization.^[2]^ HIV strikingly increases the risk of progression to active TB and the mortality associated with TB.^[3]^

Treatment success rate, it should be noted, remains lower among people living with HIV (77% globally in 2019), although there have been steady improvements over time. The number of TB deaths among people living with HIV infection was reduced to 63% in 2019 compared to 2010, against a target of 75% in 2020. While great progress has been made in achieving these targets, work remains to be done as gaps remain in coverage and quality of TB/HIV diagnosis, treatment, prevention, and care.

China is among the 30 high TB/HIV burden countries where progress is most needed to achieve the targets set in the World Health Organization End TB Strategy in the period 2021 to 2025.^[2]^ It was estimated that the number of incident TB cases in China was 780 000 in 2021, corresponding to an incidence rate of 55 (47–63) per 100,000 population, out of them 10,000 were HIV-positive.^[4]^ Although treatment guidelines to improve the treatment outcome in patients with TB/HIV co-infection have been established, TB remains one of the most common causes of death in HIV-infected patients. A previous study indicated that the overall mortality was 15.92% in TB/HIV co-infected patients in mainland China.^[5]^ Sichuan Province, where the TB/HIV co-infection prevention and control pilot project and the Global Fund project were implemented, features the largest population living with HIV in southwest China, and the prevalence of HIV among patients with TB in Sichuan has always been high. A cross-sectional study conducted in Sichuan, Guangxi, and Henan revealed that among patients with TB, 3.3% were HIV-positive.^[6]^ Therefore, as a province with a high burden of TB and acquired immune deficiency syndrome (AIDS), TB/HIV co-infection has become a serious public health problem in Sichuan Province.

A wide range of estimates for mortality and risk factors of TB/HIV co-infection has been reported in other countries. However, to the best of our knowledge, few studies have utilized large individual case-based provincial databases, especially in China. In addition, inconsistent findings from various studies support further studies to assess whether the factors reported in previous studies^[7]^ are applicable in our local setting. Therefore, it is necessary to assess whether risk factors for TB/HIV death have changed over time. This study used the large individual case-based provincial database of TB/HIV co-infected patients in Sichuan in 2020 to assess survival and factors associated with death. The results of this study can be used to develop appropriately targeted interventions and to reevaluate the clinical care management of anti-TB therapy in TB/HIV co-infected patients.

## 2. Material and methods

### 2.1. Study design and setting

A retrospective cohort study was performed on TB/HIV co-infected patients admitted to 199 designated medical institutions in Sichuan Province, a province of 83.67 million people in Southwest China,^[8]^ from January 2020 to December 2020, and the last follow-up date was December 2021. The data involving all TB/HIV co-infected patients who started TB treatment from all 21 regions of Sichuan province, were available in the National TB Surveillance System database.

### 2.2. Population

No sample size calculations were performed for this analysis. Patients were included in this study if they were diagnosed with both HIV/AIDS and *M. tb* (pulmonary TB [PTB] and extra-pulmonary TB [EPTB]) infection and initiated TB treatment between January 2020 and December 2020 at all designated TB or HIV/AIDS medical institutions in Sichuan Province. Follow-up records were reviewed every month to confirm mortality in either the HIV/AIDS or TB registration systems. For patients who died during the follow-up period, information was obtained by investigating their families. The discharged patients were followed up at the outpatient clinic. Records with missing information on treatment outcomes or dates were excluded.

We collected individual case information, such as demographics, TB microbiological test results, concurrent disease, anti-TB treatment outcomes, baseline cluster of differentiation 4 (CD4)^+^T-lymphocyte count at the time of HIV diagnosis, antiretroviral treatment (ART) initiation, and clinical status, through the electronic TB patient register and paper-based TB patient registers.

### 2.3. Variable measurement

The main outcome was death during TB treatment, irrespective of the cause.^[9]^ The outcome was classified as either death or censorship. Survival time was defined as the time between TB treatment initiation and death or censoring (time in months). Patients whose treatment outcomes were classified as cured, completed treatment, treatment failure, lost to follow-up, adverse events, and transition to drug-resistant TB were considered censored based on the last visit outcome data available in the database. Patients were followed-up until the end of their treatment or death, whichever came first. Patients who died or were alive at the end of TB treatment, the date of death, or the date of the end of TB treatment were considered the endpoint of the follow-up.

Independent variables included sex (male and female), age categorized as 0 to 20 years, 21 to 40 years, 41 to 60 years, and >60 years, ethnicity (Han, Yi, and other minorities), and occupation (unemployed, employed, and others). Similarly, the level of health facilities where patients received TB treatment was categorized into city-level hospitals and county-level hospitals. Referral types were classified as self-referral, tracing by health facilities, and other referrals. The patients with confirmed TB were classified as new and re-treatment (relapse, treatment after failure, treatment after loss to follow-up). Anatomical sites of TB were classified as P (disease affecting the lungs only) and EP (disease affecting the lungs and other organs). TB results were grouped into bacteriologically confirmed (sputum smear, culture, or molecularly confirmed) or bacteriologically unconfirmed (smear-negative, physician-confirmed through other means). Clinical severity (severely ill or not severely ill). ART status was classified as started ART or not started, while the baseline CD4^+^T-lymphocyte count was grouped into <200 cells/μL, ≥200 cells/μL, and unknown.

In China, a diagnosis of *M. tb* was made based on the combined evaluation of clinical, radiological, histopathological, and laboratory features of the patients according to the protocol established by the National Tuberculosis Prevention and Control Program (2008): sample smears/cultures positive or sample smears/cultures negative but met all three of the following clinical criteria: symptoms consistent with TB; chest X-ray suggestive of TB; and positive anti-TB antibody response. A patient was confirmed to be HIV-infected if HIV screening is positive, and then 2 re-tests are carried out. If one of the 2 re-tests is positive, further antibody confirmation or nucleic acid testing is required to confirm HIV infection; or nucleic acid test is positive twice at different times; or if the virus is directly isolated positive. For patients with TB, hospitals provide directly observed treatment strategy in both adult and pediatric age groups with free drugs like 2-month intensive phase with rifampicin, isoniazid, pyrazinamide, and ethambutol (2HRZE) and 4-month continuation phase with rifampicin and isoniazid (4RH).

### 2.4. Data analysis and statistics

IBM SPSS version 17.0 (IBM SPSS Inc., Armonk, NY) was used for data entry and analysis. Categorical sociodemographic and clinical variables were summarized as frequencies and proportions and compared using Pearson chi-squared test and Fisher exact test. We calculated the overall and covariate-specific TB/HIV co-infection mortality rates per 1000 person-months (pm) using the Kaplan–Meier estimator. Kaplan–Meier curves were used to assess univariate analysis and survival probabilities, and a log-rank test was used to test statistical significance differences between the survival curves. We performed multivariate analyses using Cox proportional-hazard regression models, and factors with a *P* value of ≤.2 in the univariate analysis were considered potential risk factors and were included in the multivariable model. Hazard ratios (HRs) and respective 95% confidence intervals (CIs) were reported. All tests were 2-sided, and statistical significance was set at *P* < .05.

## 3. Results

In 2020, 849 TB/HIV co-infections were confirmed and registered in the TB surveillance information management system of Sichuan Province. Of these, 21 were excluded from the study because they were not in contact after discharge. Finally, 828 co-infection cases were identified for the analysis. The included and excluded records did not differ in terms of sociodemographic and clinical characteristics. 44.9% of patients were aged between 21 and 40 years. Males accounted for 659 (79.6%) of the study population. The majority of patients (n = 434, 52.4%) were of Han nationality. Most patients 749 (90.5%) were unemployed. Of all co-infections, 92.4% (765) were newly diagnosed and 740 (89.4%) had PTB only. More than half of the patients 461 (55.7%) had bacteriologically unconfirmed TB (Table 1).

**Table 1:** Social-demographic and clinical characteristics of TB/HIV patients.

Characteristics	Number	Percentage
Gender		
Female	169	20.4
Male	659	79.6
Age group (yr)		
0–20	23	2.8
21–40	372	44.9
41–60	301	36.4
>60	132	15.9
Ethnicity		
Han	434	52.4
Yi	379	45.8
Others	15	1.8
Occupation		
Unemployed	749	90.5
Employed	54	6.5
Unknown	25	3.0
Referral type		
Self-referral	228	27.6
Tracing	586	70.9
Others	12	1.5
Patient type		
New	765	92.4
Re-treatment	63	7.6
Health facility level		
County-level	780	94.2
Municipal	48	5.8
Anatomical site of TB		
PTB	740	89.4
PTB and EPTB	88	10.6
TB results		
Bacteriologically unconfirmed	461	55.7
Bacteriologically confirmed	367	44.3
Severely ill		
No	774	93.5
Yes	54	6.5
ART initiation		
Yes	481	58.1
No	347	41.9
CD4^+^T-lymphocyte count (cells/μL)		
≥200	217	26.2
<200	167	20.2
Unknown/missing	444	53.6

ART = anti-retroviral therapy, CD4 = cluster of differentiation 4, EPTB = extra-pulmonary tuberculosis, HIV = human immunodeficiency virus, PTB = pulmonary tuberculosis, TB = tuberculosis.

During a 12-month follow-up period, 5673 pm were observed. Fort-four (5.31%) participants died within 1 year, and the crude mortality was estimated at 7.76 per 1000 pm. More than half of the deaths (52.27%) occurred during the initial 3 months of anti-TB treatment. Mortality in the 3rd month since starting treatment and the 6th month since starting treatment were 315.07 and 11.94 per 1000 pm respectively. TB/HIV co-infected patients who visited city-level health facilities, were severely ill, had both PTB and EPTB coinfection, did not receive ART, and with a baseline CD4^+^T-lymphocyte count <200 cells/μL had the highest mortality rate. The mortality rates of the different covariates are shown in Table 2.

**Table 2:** Mortality of TB/HIV co-infected patients.

Characteristics	Person months (pm)	Number of deaths	Mortality per 1000 pm
Crude mortality	5673	44	7.76
Mortality since TB treatment started			
3rd mo	73	23	315.07
6th mo	3183	38	11.94
12th mo	5673	44	7.76
Gender			
Female	1106	5	4.52
Male	4567	39	8.54
Age group (yr)			
0–20	147	0	0.00
21–40	2525	20	7.92
41–60	2117	17	8.03
>60	884	7	7.92
Ethnicity			
Han	3017	30	9.94
Yi	2528	13	5.14
Others	128	1	7.81
Occupation			
Unemployed	5182	41	7.91
Employed	341	2	5.87
Unknown	150	1	6.67
Referral type			
Self-referral	1640	11	6.71
Tracing	3957	33	8.34
Others	69	0	0.00
Patient type			
New	5151	40	7.77
Re-treatment	522	4	7.66
Health facility level			
County-level	5336	38	7.12
City-level	337	6	17.80
Anatomical site of TB			
PTB	4937	33	6.68
PTB and EPTB	736	11	14.95
TB results			
Bacteriologically unconfirmed	3159	18	5.70
Bacteriologically confirmed	2514	26	10.34
Severely ill			
No	5277	39	7.39
Yes	396	5	12.63
ART initiation			
Yes	3297	16	4.85
No	2376	28	11.78
CD4^+^T-lymphocyte count (cells/μL)			
≥200	1474	4	2.71
<200	1107	14	12.65
Unknown/missing	3092	26	8.41

ART = anti-retroviral therapy, CD4 = cluster of differentiation 4, EPTB = extra-pulmonary tuberculosis, HIV = human immunodeficiency virus, PTB = pulmonary tuberculosis, TB = tuberculosis.

**Figure 1.**
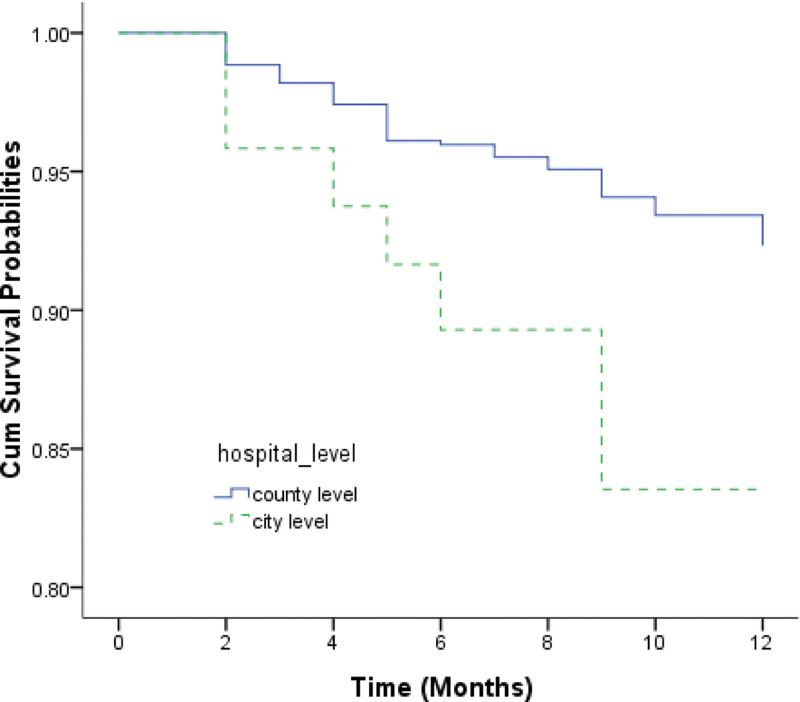
Kaplan–Meier survival estimate shows the survival probabilities among TB/HIV co-infected patients under county-level and city-level hospitals options. HIV = human immunodeficiency virus, TB = tuberculosis.

**Figure 2.**
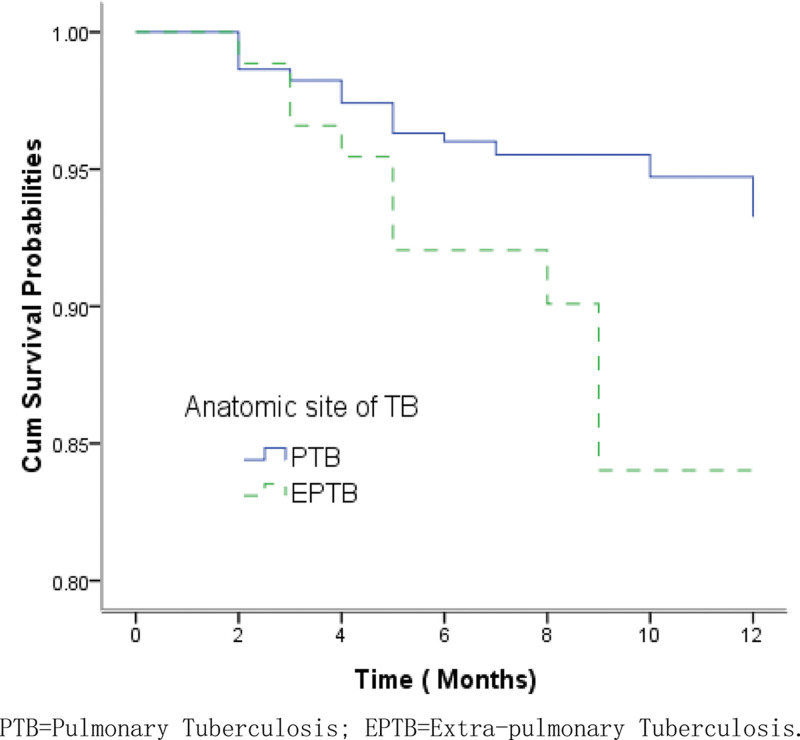
Kaplan–Meier survival estimate shows the survival probabilities among pulmonary TB and extra pulmonary TB patients with HIV co-infection. HIV = human immunodeficiency virus, TB = tuberculosis.

**Figure 3.**
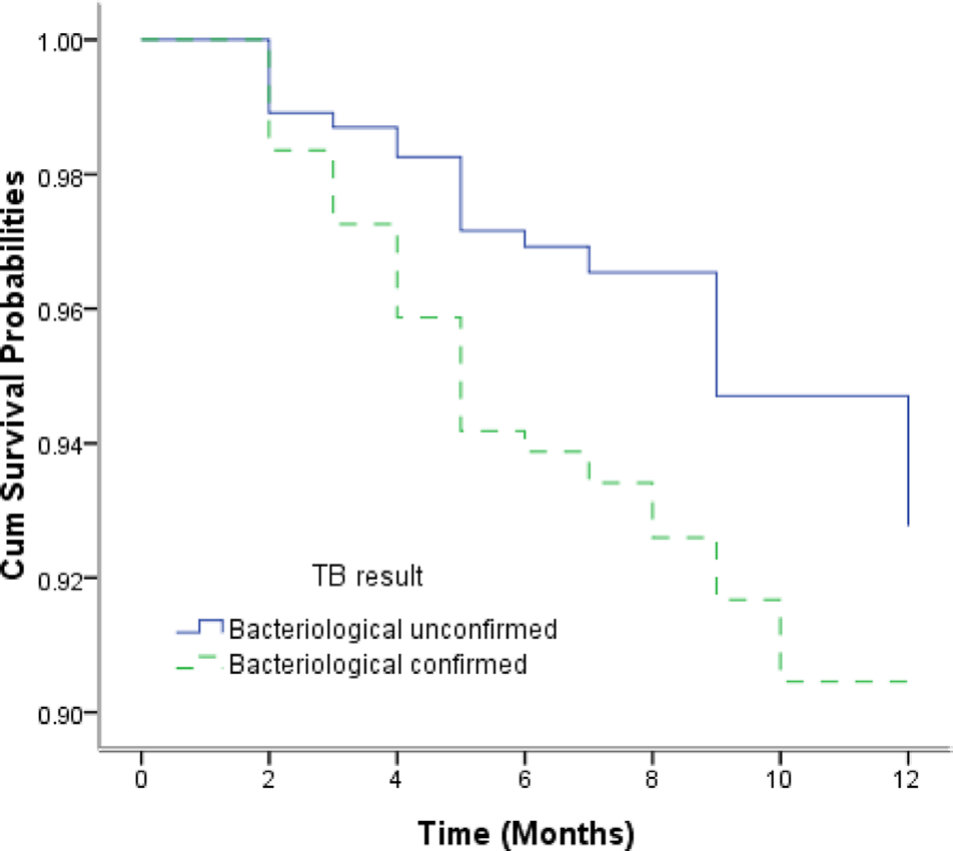
Kaplan–Meier survival estimate shows the survival probabilities among bacteriological unconfirmed and confirmed TB/HIV co-infections. HIV = human immunodeficiency virus, TB = tuberculosis.

**Figure 4.**
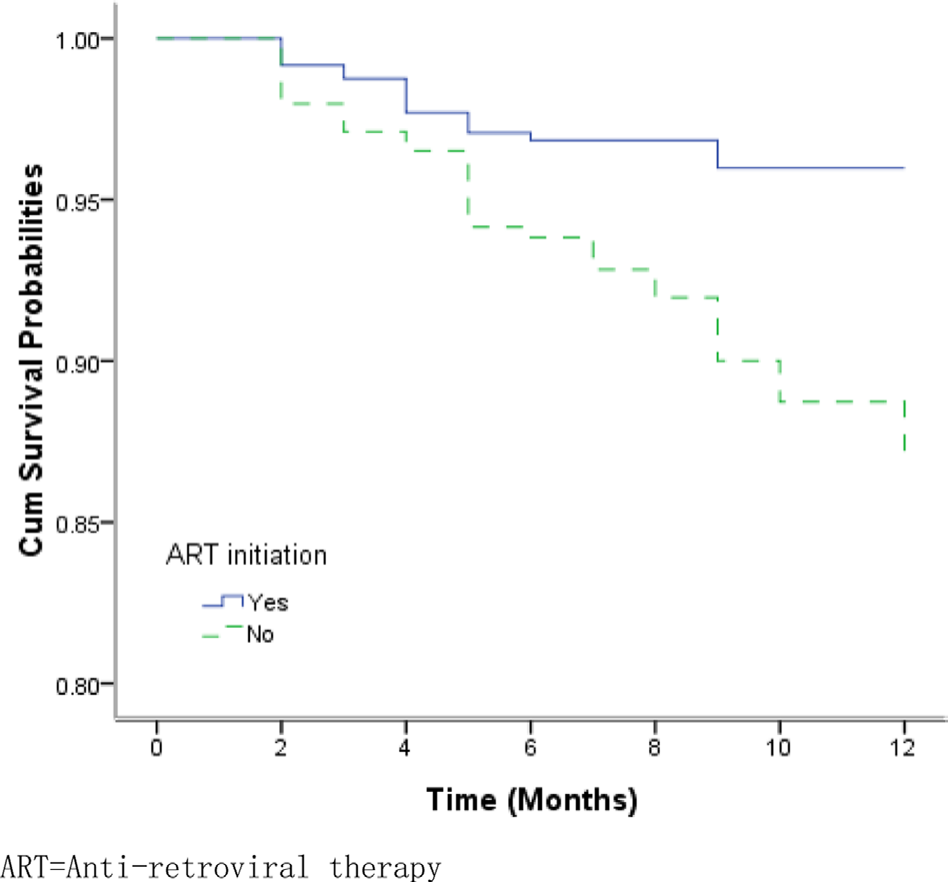
Kaplan–Meier survival estimate shows the survival probabilities among patients on ART or not. ART = antiretroviral therapy.

**Figure 5.**
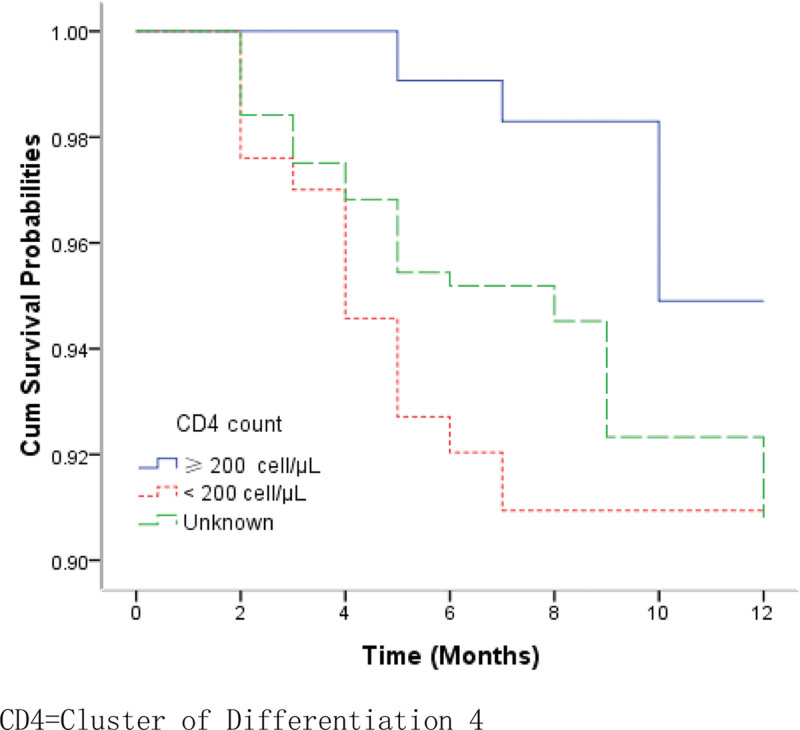
Kaplan–Meier survival curves show the survival probabilities among patients with CD4 + T-lymphocyte count ≥ 200 cell/μL, <200 cell/μL, and unknown. CD4 = cluster of differentiation 4.

The overall survival probabilities of TB/HIV co-infected patients were estimated to be 97.20%, 95.16%, and 91.75% at the 3rd, 6th, and 12th months respectively. Figure 1 shows the Kaplan–Meier estimates for the group of patients who accessed services at county-level health facilities and the group of those at city-level hospitals. The median survival times for the 2 groups were 19.9 months and 12.5 months, respectively. The log-rank test revealed that there were significantly lower survival probabilities in the group of patients receiving TB treatment at city-level hospitals than in those receiving treatment at county-level health facilities (*χ*^2^ = 4.699, *P* = .030). EP and PTB patients infected with HIV had significantly lower survival probabilities compared to the group of PTB patients with HIV co-infection (*χ*^2^ = 7.038, *P* = .008) (Fig. 2). Patients with bacteriologically confirmed TB/HIV co-infected had significantly lower survival probabilities compared to patients with bacteriologically unconfirmed (*χ*^2^ = 3.875, *P* = .049) (Fig. 3). The log-rank test also revealed that there were significantly lower survival probabilities among TB/HIV co-infections who did not start ART during TB treatment than among those who started ART (*χ*^2^ = 8.434, *P* = .004) (Fig. 4). Kaplan–Meier curves showed significantly lower survival probabilities among TB/HIV co-infections with a low baseline CD4^+^T-lymphocyte count (*χ*^2^ = 8.439, *P* = .015) (Fig. 5). Univariate analysis failed to find significant correlations between age group, sex, ethnicity, occupation, type of referral, patient type, the severity of illness, and survival probability of TB/HIV co-infected patients. Therefore, factors with *P* < .2 in univariate analysis were included in the Cox regression model (Table 3).

**Table 3:** Univariate and multivariate analysis on the risk factors of mortality among TB/HIV co-infected patients.

Variable	Univariate	Multivariate	
	*P* value	Hazard ratio (95% CI)	*P* value
Gender			
Female	0.159	Reference	
Male		1.711 (0.662–4.425)	.268
Age group (yr)			
0–20	0.744		
21–40			
41–60			
>60			
Ethnicity			
Han	0.124	Reference	
Yi		0.865 (0.420–1.781)	.694
Others		1.271 (0.165–9.768)	.818
Occupation			
Unemployed	0.883		
Employed			
Unknown			
Referral type			
Self-referral	0.635		
Tracing			
Others			
Patient type			
New	0.985		
Re-treatment			
Health facility level			
County-level	0.03	Reference	
City-level		1.577 (0.627–3.963)	.333
Anatomical site of TB			
PTB	0.008	Reference	
PTB and EPTB		2.073 (1.020–4.216)	.044
TB results			
Bacteriologically unconfirmed	0.049	Reference	
Bacteriologically confirmed		1.593 (0.862–2.945)	.137
Severely ill			
No	0.252		
Yes			
ART initiation			
Yes	0.004	Reference	
No		2.534 (1.213–5.294)	.013
CD4 count (cells/μL)			
≥200	0.015	Reference	
<200		3.505 (1.104–11.129)	.033
Unknown/missing		1.596 (0.516–4.942)	.417

ART = anti-retroviral therapy, CD4 = cluster of differentiation 4, CI = confidence interval, EPTB = extra-pulmonary tuberculosis, HIV = human immunodeficiency virus, PTB = pulmonary tuberculosis, TB = tuberculosis.

After adjusting for potential confounders and other variables, the independent risk factors for TB/HIV co-infection mortality were as follows: having both EP and PTB coinfection (HR = 2.073, 95% CI = 1.020–4.216, *P* = .044), not initiated ART (HR = 2.534, 95% CI = 1.213–5.294, *P* = .013), and with the baseline CD4^+^T-lymphocyte count < 200 cells/μL (HR = 3.505, 95% CI = 1.104–11.129, *P* = .033) (Table 3).

## 4. Discussion

Finally, our study included 828 TB/HIV co-infected patients. To our knowledge, this is the first large-scale study in Sichuan that evaluated the mortality of patients with TB/HIV co-infection. Despite the implementation of national free ART for more than a decade,^[10]^ the overall mortality rate was 7.76 per 1000 pm during TB treatment among TB/HIV co-infections in our study. This is less than those of cohort studies conducted in one health facility in Tanzania, East Africa (11.34 per 1000 pm),^[11]^ 40 rural clinics in South Africa (10.1 per 100 person-years),^[12]^ and in Kampala Uganda (16.0 per 100 person-years).^[13]^ Similarly, the proportion of deaths among TB/HIV co-infected patients was 5.31%, which is much less than that reported in previous studies conducted in Shanghai, China (17.7%),^[7]^ and in a hospital in Beijing, China (18.41%).^[5]^ The difference in the proportion of deaths in this study could be attributed to differences in the study population between this study and other studies. The majority of previous research has hardly used the database of all TB-designated hospitals in a whole province like ours. The high mortality among people with TB/HIV co-infection in the initial 3 months of TB treatment after TB diagnosis in this study was coincident with the study conducted in a previous study in Eastern Europe (61.4 per 100 person-years of follow-up).^[14]^ This result suggests that early diagnosis of TB in people with HIV infection and timely use of anti-TB therapy following TB diagnosis may improve outcomes and possibly even minimize the proportion of deaths in TB/HIV co-infected patients.

Disseminated TB affects multiple organs, resulting in high mortality, but often presents nonspecifically, which may impede prompt diagnosis.^[15,16]^ Our Cox proportional hazard model also demonstrated that death was more likely to occur in EP and PTB (TB detected in both sputa and at least 1 EP specimen) than in patients co-infected with HIV/PTB (TB detected in sputum only). This is consistent with research conducted by Schutz et al, who identified that EP and PTB, sepsis syndrome, and rifampicin resistance were associated with mortality.^[17]^ TB/HIV co-infection often has a nonspecific clinical presentation (i.e., without cough) and a high proportion of sputum smear-negative and radiographically nonspecific diseases. Therefore, rapid and sensitive TB detection in people with HIV infection should be considered a top priority to improve treatment outcomes and reduce mortality.

We also found that a lower baseline CD4 count at the time of HIV diagnosis was associated with mortality in patients with TB/HIV coinfection. This result is consistent with the literature in South Africa and Brazil^[18,19]^ but is contrary to previous studies^[5,20,21]^ conducted in other parts of China. CD4^+^T lymphocytes produce interferon-γ, which is important in the immune response against TB.^[22]^ CD4^+^lymphocytopenia is indicative of severe disease and impaired cell-mediated immune responses against TB.^[23]^ Indeed, levels of immune activation are thought to be the best predictor of progression from HIV infection to AIDS and even death, independent of the HIV viral load.^[24]^ Tuberculosis can occur at any CD4 cell level; however, when CD4 levels are low, infection with other pathogens progresses more rapidly, and an abnormal increase in HIV replication in a TB combination accelerates the deterioration of the disease. As a result, when CD4 cell levels are low, in addition to active highly active ART and anti-TB therapy, the patient’s immune system should be improved to enhance the treatment of other pathogens and reduce the occurrence of death.

Similar to previous studies,^[25,26]^ our multivariate model demonstrated that ART initiation was significantly associated with higher odds of mortality among HIV-positive TB patients. Based on the available evidence, the European AIDS clinical society recommends the following: “ART should be started as soon as possible (within 2 weeks of initiating TB treatment) regardless of CD4 count. However, if TB meningitis signs and symptoms are present ART initiation may be delayed. Be aware of immune reconstitution syndrome reaction in persons starting ART at low CD4 count levels and with early initiation of ART.”^[27]^ Therefore, more attention should be paid to TB/HIV co-infected patients with TB meningitis or immune reconstitution syndrome. ART and anti-TB treatment should be carried out with caution, and attention should be paid to the adverse reactions during treatment. Nevertheless, most HIV-infected patients in many developing countries still cannot access ART, primarily because of economic barriers.^[28]^ Even if the Chinese government initiated the National Free Antiretroviral Treatment Program in 2003,^[10]^ it was impossible for every patient infected with HIV in China to be covered by this program to initiate ART after diagnosis. Moreover, some patients are hesitant to undergo ART until they develop severe opportunistic infections that are difficult to survive. This is also supported by our study, in which patients who received ART accounted for only 58.1%. Therefore, patients should be informed by the healthcare provider about the best available evidence regarding the rapid start of ART and the benefit of ART during medical consultations. ART interruption is expected to be associated with failure to reconstitute the immune system, as discussed above.^[29]^ This indicates that interventions, such as integrating or optimizing ART and TB care, would facilitate ART adherence and improve TB outcomes. Ultimately, holistic, high-quality, person-centered care is needed for patients with TB/HIV coinfection throughout the cascade of care, which should address biomedical, socioeconomic, and psychological barriers.^[30]^

Apart from these significant findings, our study had several limitations. First, because this study used an existing database, only limited demographic and clinical factors were included to explain the mortality risk among patients with TB/HIV coinfection. Socioeconomic and behavioral risk factors were not considered. Second, since all data are stored in an existing database, it was not feasible to capture the missing data on some variables, such as the baseline CD4 T-cell counts. Therefore, missing information may have biased risk estimates. Bias is always a concern in secondary data sources.

## 5. Conclusion

In conclusion, the mortality rate of TB/HIV coinfection is low in reference hospitals or health facilities in Sichuan. However, more effort is needed to achieve the End TB strategy. Screening of TB/HIV coinfection with a sensitive and effective method and receiving ART with an optimal combination of antiretroviral and anti-TB therapy as early as possible are essential for the survival of patients with TB/HIV coinfection, and immunomodulatory therapy is recommended to prevent EPTB and help prolong life. These findings provide pathophysiological insights into the underlying causes of mortality and could be used to inform the development of novel treatment strategies and methods to risk-stratify patients to appropriately target novel interventions.

## Acknowledgments

The authors thank Dr Zhang and Dr Kang for their linguistic assistance.

## Author contributions

**Data curation:** Jing Li, Yin Zhong.

**Investigation:** Jing Li, Yin Zhong, Yang Song.

**Supervision:** Ni Yang, Jinge He, Chuang Chen.

**Writing – review & editing:** Ni Yang, Jinge He.
